# Standard versus accelerated initiation of renal replacement therapy in acute kidney injury (STARRT-AKI): study protocol for a randomized controlled trial

**DOI:** 10.1186/1745-6215-14-320

**Published:** 2013-10-05

**Authors:** Orla M Smith, Ron Wald, Neill KJ Adhikari, Karen Pope, Matthew A Weir, Sean M Bagshaw

**Affiliations:** 1 Critical Care Department, St. Michael’s Hospital, 30 Bond Street, Toronto, ON M5B1W8, Canada; 2 Keenan Research Centre, Li KaShing Knowledge Institute, St. Michael’s Hospital, 30 Bond Street, Toronto, ON M5B1W8, Canada; 3 Division of Nephrology, St. Michael’s Hospital, 30 Bond Street, Toronto, ON M5B1W8, Canada; 4 Department of Medicine, University of Toronto, 30 Bond Street, Toronto, ON M5B1W8, Canada; 5 Department of Critical Care Medicine, Sunnybrook Health Sciences Centre and University of Toronto, Room D1.08, 2075 Bayview Avenue, Toronto, ON M4N 3M5, Canada; 6 Applied Health Research Centre, Li KaShing Knowledge Institute, St. Michael’s Hospital, 30 Bond Street, Toronto, ON M5B1W8, Canada; 7 Division of Nephrology, Western University, London, ON N6A 3K7, Canada; 8 University Hospital, ALL-139A 339 Windermere Road, London, ON N6A 5A5, Canada; 9 Division of Critical Care Medicine, Faculty of Medicine and Dentistry, University of Alberta, 3C1.12 Walter C. Mackenzie Centre, 8440-112 St, Edmonton, NW RILF3 T6G2B7, Canada

**Keywords:** Acute kidney injury, Critical illness, Dialysis, Hemodialysis, Renal replacement therapy, Critical care

## Abstract

**Background:**

Acute kidney injury is a common and devastating complication of critical illness, for which renal replacement therapy is frequently needed to manage severe cases. While a recent systematic review suggested that “earlier” initiation of renal replacement therapy improves survival, completed trials are limited due to small size, single-centre status, and use of variable definitions to define “early” renal replacement therapy initiation.

**Methods/design:**

This is an open-label pilot randomized controlled trial. One hundred critically ill patients with severe acute kidney injury will be randomly allocated 1:1 to receive “accelerated” initiation of renal replacement therapy or “standard” initiation at 12 centers across Canada. In the accelerated arm, participants will have a venous catheter placed and renal replacement therapy will be initiated within 12 hours of fulfilling eligibility. In the standard initiation arm, participants will be monitored over 7 days to identify indications for renal replacement therapy. For participants in the standard arm with persistent acute kidney injury, defined as a serum creatinine not declining >50% from the value at the time of eligibility, the initiation of RRT will be discouraged unless one or more of the following criteria are fulfilled: serum potassium ≥6.0 mmol/L; serum bicarbonate ≤10 mmol/L; severe respiratory failure (PaO_2_/FiO_2_<200) or persisting acute kidney injury for ≥72 hours after fulfilling eligibility. The inclusion criteria are designed to identify a population of critically ill adults with severe acute kidney injury who are likely to need renal replacement therapy during their hospitalization, but not immediately. The primary outcome is protocol adherence (>90%). Secondary outcomes include measures of feasibility (proportion of eligible patients enrolled in the trial, proportion of enrolled patients followed to 90 days for assessment of vital status and the need for renal replacement therapy) and safety (occurrence of adverse events).

**Discussion:**

The optimal timing of renal replacement therapy initiation in patients with severe acute kidney injury remains uncertain, representing an important knowledge gap and a priority for high-quality research. This pilot trial is necessary to establish protocol feasibility, confirm the safety of participants and obtain estimated events rates for design of a large definitive trial.

**Trial registration number:**

NCT01557361

## Background

Acute kidney injury (AKI) is a common complication in critically ill patients. Among those with severe AKI, 50 to 70% will receive renal replacement therapy (RRT) [1]. RRT increases the complexity and costs of care and is associated with death, with case-fatality rates commonly exceeding 60% [1,2], and dialysis dependence among survivors [2-4].

Methods of providing RRT to patients in the ICU vary widely, reflecting uncertainty surrounding many aspects of this therapy [5,6]. It is generally understood that life-threatening AKI complications, such as severe hyperkalemia, profound acidosis and fluid overload resulting in respiratory failure, are absolute indications for RRT. However, outside of these indications, the optimal time to start RRT in critically ill patients with severe AKI remains unknown. Early initiation of RRT has the intuitive appeal of avoiding life-threatening AKI complications; however, RRT is not without complications and costs [7,8], and in the setting of chronic dialysis for end-stage renal disease, no evidence of benefit was found with earlier start times [9].

We recently performed a systematic review of the effect of RRT timing on mortality in adult critically ill patients, and included 15 studies published between 1999 and 2010 [10]. In a pooled analysis limited by low methodological quality of the included studies and important statistical heterogeneity, early (versus later) initiation of RRT was associated with a significantly reduced odds of death (odds ratio 0.45, 95% CI 0.28, 0.72). The largest published randomized controlled trial (RCT) (n=106) of RRT timing found no difference in mortality; however, it was underpowered to detect a clinically implausible absolute difference in mortality of less than 40% [11]. For comparison, the large multi-cente rtrial, Randomized Evaluation of Normal versus Augmented Level Renal Replacement Therapy (RENAL),enrolled 1,508 critically ill patients with severe AKI and was powered to detect a minimal difference in 90-day mortality of 7.5% [12].

Given the limitations of published studies, a large and methodologically rigorous RCT is needed to evaluate the impact on survival and renal recovery of earlier versus current usual criteria for RRT initiation in critically ill adults with severe AKI. This question has also been identified as a top research priority by the Acute Kidney Injury Network [13] and endorsed by intensivists and nephrologists [14].

Our objective is to conduct a Canadian multi-centerRCT in critically ill patients with severe AKI, comparing the effect of accelerated versus standard RRT initiation on 90-day mortality and dialysis dependence among 90-day survivors. To support this trial, we propose to perform a pilot trial to evaluate protocol adherence, estimate recruitment rates, confirm that meaningful separation in the timing of RRT between treatment arms can be achieved, and evaluate safety.

## Methods/design

### Design

The design is an open-label, multi-center, pilot, randomized controlled trial of accelerated versus standard initiation of RRT initiation in critically ill patients with severe AKI.

### Setting

The setting is the ICU at 12 academic hospitals across Canada. Site investigators are listed in Additional file 1.

### Population

Eligible participants will fulfill all inclusion and no exclusion criteria (see Additional file 2).

### Inclusion criteria

The inclusion criteria are designed to identify a population of critically ill adults with severe AKI who have an increased likelihood of receiving RRT during their hospitalization, but who do not have urgent indications for the initiation of RRT at the time of fulfilling eligibility criteria. All six criteria must be fulfilled at the time of screening:

1. Age ≥18 years.

2. Admission to an ICU.

3. Evidence of kidney dysfunction (serum creatinine above the upper threshold of normal for commercially available laboratory assays (women ≥100 μmol/L; men ≥130 μmol/L)

4. Evidence of severe AKI based on at least two of the following three criteria:

i. A 2-fold increase in serum creatinine during hospitalization or from a known pre-hospitalization baseline with current creatinine no less than 25 μmol/L below peak (consistent with the Risk, Injury, Failure, Loss, End-stage renal failure (RIFLE) INJURY category of AKI);

i. Oliguria as defined by urine output <6 mL/kg over the preceding 12 hours (consistent with RIFLE category of AKI);

i. If only one of two above criteria for severe AKI are met and all other eligibility criteria are fulfilled, a whole-blood Cardiorenal Neutrophil Gelatinase-Associated Lipocalin™ (NGAL)(Alere Inc., San Diego, CA, USA) ≥400 ng/mL.

5. Likelihood that an absolute indication for RRT will not arise in the subsequent 24 hours based on the most recent blood laboratory values:

i. Serum potassium ≤5.5 mmol/L, and

i. Serum bicarbonate ≥15 mmol/L.

6. Central venous pressure ≥8 mmHg. The rationale for this criterion is to ensure adequate volume resuscitation and mitigate enrollment of patients with hypovolemia or AKI that will readily improve with fluid administration.

### NGAL testing for eligibility

NGAL is a novel biomarker that is upregulated early in the course of AKI [15]. Whole blood (0.25 to 0.50 mL) will be tested using a fluorescence immunoassay that operates on the Triage™ platform (Alere Inc.) and yields quantitative results within 15 to 20 minutes. NGAL may detect AKI prior to excursions in the serum creatinine [16,17] and has been found useful for discriminating between patients whose elevated serum creatinine values are due to established acute kidney damage (that is, the target population for this trial) compared to chronic kidney disease or uncomplicated volume contraction [18]. A recent meta-analysis also found that plasma NGAL concentrations predict worsening AKI and the receipt of RRT with an estimated area under the receiver operator curve of 0.78 [19]. We chose a cutoff value for NGAL of 400 ng/mL based on data from recently published studies [20,21] which found the operating characteristics for a serum NGAL of 417 ng/mL for predicting the need for RRT (that is, development of RIFLE-Failure AKI) to be as follows: sensitivity 0.70; specificity 0.90; positive predictive value 0.40; and negative predictive value 0.97. The high negative predictive value identifies this threshold as a specific predictor for subsequently receiving RRT [20,21].

### Exclusion criteria

1. Lack of commitment to ongoing life support, including RRT.

2. Presence of a drug overdose that necessitates initiation of RRT.

3. Any RRT within the previous 2 months.

4. Presence or clinical suspicion of renal obstruction, rapidly progressive glomerulonephritis, vasculitis, or acute interstitial nephritis.

5. Known pre-hospitalization advanced chronic kidney disease, defined by an estimated glomerular filtration rate <30 mL/minute/1.73 m^2^, or chronic RRT.

6. Kidney transplant within the past 365 days.

7. Time since first doubling of serum creatinine has exceeded 48 hours.

8. Clinician(s) caring for patient believe(s) that immediate RRT is absolutely mandated.

9. Clinician(s) caring for patient believe(s) that deferral of RRT initiation is mandated.

10. Patient or substitute decision maker is unable to provide consent within 12 hours of determination of study eligibility.

### Eligible, non-randomized patients

Eligible non-randomized patients will be those who meet all inclusion criteria but are not included in the trial for the following reasons:

1. A doubling of serum creatinine that has exceeded 48 hours at the time of screening (exclusion 7);

2. Clinician(s) belief that either immediate RRT is absolutely mandated (exclusion 8) or deferral of RRT initiation (exclusion 9) is absolutely mandated; or

3. Refusal of consent or inability to obtain consent within 12 hours of patient meeting eligibility criteria (exclusion 10).

We will document reasons leading to non-inclusion of these otherwise eligible patients. For these patients, we will collect data on kidney function at baseline and at the time of eligibility, severity of illness at the time of eligibility, receipt of RRT in hospital and need for RRT at hospital discharge, and vital status at ICU and hospital discharge. The collection of these data will enable comparisons with randomized patients to assess threats to the generalizability of the trial results.

### Screening process

Research coordinators will screen patients in each study ICU for eligibility twice each weekday, in the morning and afternoon. Given the dynamic nature of the inclusion criteria, individuals who are not initially eligible for the trial will be re-screened each day. Research coordinators will discuss all patients who meet inclusion criteria, without an exclusion criterion, with the attending intensivist and, if applicable, the attending nephrologist.

### Ethics and consent

The Health Research Ethics Boards (HREB) at the University of Alberta (Edmonton) and St. Michael’s Hospital (Toronto), along with those at each participating site, reviewed and approved the study (see Additional file 3). Research coordinators will approach eligible patients or their substitute decision maker (SDM), in the event of patient incapacity, for informed consent (see Additional file 4). If the SDM provides consent but the patient becomes capable prior to the end of the follow-up period, the research coordinator will seek consent directly from the patient, who will have the option of withdrawing from the study. If an SDM is contactable by telephone but is not able to come in person to sign the consent form, steps will be taken to obtain telephone consent.

At four sites where this study has Research Ethics Board (REB) approval, patients who are eligible but incapable of providing consent and for whom an SDM is not available may be randomized through a process of deferred consent (see Additional file 5). In this circumstance, the research coordinator will endeavor to locate an SDM after patient enrollment, and ask the SDM to affirm or withdraw consent for the patient’s participation. In addition, once the participant regains capacity, he/she will be asked to affirm or withdraw consent for the trial.

### Co-enrollment

Patients recruited to the study, STtandard versus Accelerated initiation of Renal Replacement Therapy in Acute Kidney Injury (STARRT-AKI), may also be enrolled in other studies provided that the steering committee has reviewed the protocol and determined that there is no interference with the timing of RRT initiation mandated by the STARRT-AKI protocol. Prior survey findings have shown clinical researchers’ support for trial co-enrollment of critically ill patients [22].

### Randomization process

Participants will be randomly allocated in a ratio of 1:1, stratified by study center using variable block sizes undisclosed to site study personnel, to accelerated or standard initiation of RRT. The study statistician at the methods centre (Applied Health Research Centre (AHRC), St. Michael’s Hospital, Toronto, Canada) will generate the random allocation sequence using computer software. Randomization information for each site will be contained in locally held sequentially numbered, opaque, sealed envelopes. Envelopes are to be opened in numeric sequence.

### Interventions

Accelerated RRT initiation (experimental arm):a catheter will be placed by the treating ICU or Nephrology team and RRT initiated as soon as possible, and within 12 hours of eligibility. This 12-hour window includes the time needed to obtain consent.

Standard RRT initiation (control arm):the study team will follow patients for 7 days to identify potential indications for RRT. For patients with persistent AKI (serum creatinine has not declined by more than 50% from value at time of eligibility), the protocol discourages RRT initiation unless one or more of the following criteria are met:

1. Serum potassium ≥6.0 mmol/L, or

2. Serum bicarbonate ≤10 mmol/L, or

3. Severe respiratory failure, defined as arterial partial pressure of oxygen(PaO_2_)/inspired oxygen fraction(FiO_2_)<200 and bilateral infiltrates on the chest radiograph, or

4. Persistent AKI (as defined above) 72 hours after eligibility.

Notwithstanding these recommendations, RRT in the control arm may be commenced at anytime, provided >12 hours have elapsed since the patient met eligibility criteria, at the discretion of the attending physician(s). This will not be considered a protocol deviation. If this occurs, the physician will be asked to specify the primary reason for initiating RRT on that day. If RRT is initiated within 12 hours of eligibility, the clinician will also be asked to provide primary reason(s) for this protocol violation.

### Renal replacement therapy delivery

Other than the study intervention (that is, differential timing of RRT initiation), all RRT delivered to patients in both treatment arms will follow current clinical practice guidelines [23]. The three commonly used RRT modalities may be used in this study: intermittent hemodialysis (IHD), sustained low efficiency dialysis (SLED), and continuous renal replacement therapy (CRRT). These are summarized in Table 1. The initial RRT modality will be guided by hemodynamic status at the time the patient is ready to start RRT using the cardiovascular component of the Sequential Organ Failure Assessment score (SOFA_CV_) [24]. Following the initiation of RRT, modality switches will be guided by the patient’s hemodynamic profile, as done in a recent large trial [25].

**Table 1:** Delivery of renal replacement therapy in the trial

	**Intermittent hemodialysis**	**Sustained low-efficiency dialysis**	**Continuous renal replacement therapy**
**Patient hemodynamic profile**	Off all vasopressors and inotropes (SOFA_cv_ <2)	Receiving any dose of vasopressor or inotrope (SOFA_cv_ ≥2)	Receiving any dose of vasopressor or inotrope (SOFA_cv_ ≥2)
**Minimum session duration (hrs)**	3	8	24
**Minimum frequency**	3 times/week	3 times/week	NA
**Blood flow target (mL/min)**	200 to 400	200 to 300	100 to 250
**Dialysate flow (mL/min)**	500 to 800	200 to 400	NA
**Total effluent (mL/kg/hr)**	NA	NA	≥25
**Anticoagulation options**	Heparin	Heparin	Heparin
	None	None	None
			Regional citrate anticoagulation
**Fluid removal rate**	To be determined by the nephrologist and/or critical care physician		

This table outlines guidelines for renal replacement therapy prescription by modality for randomized participants. NA, not applicable; SOFA_cv_, cardiovascular component of the Sequential Organ Failure Assessment score [22].

### Cessation of renal replacement therapy

RRT may be discontinued due to:

1. Withdrawal of renal support in the context of a change in the overall goals of care for the patient, or

2. Kidney function recovery with no need for RRT, assessed at least 48 hours after the last RRT session and defined by:

i. Decline in serum creatinine by ≥50 μmol/L on two blood tests separated by >12 hours with no RRT during the 48 hours from the last RRT session to the time of assessment; or

i. Urine output >6 mL/kg during any 12-hour period during the 48-hour period of evaluation, with most recent serum potassium <5.5 mmol/L and most recent serum bicarbonate >18 mmol/L.

### Co-interventions

The administration of volume expanders, inotropes, and vasopressors for hemodynamic support will be at the discretion of the ICU team. Vital signs and routine blood work will be performed and documented as per usual practice.

### Monitoring and follow-up

We will collect data on all RRT treatments, adverse events and serious adverse events (defined below) for 14 days after randomization. We will determine vital status and need for RRT among survivors at 90 days following randomization through chart review and direct contact with patients or SDMs, if necessary.

### Primary and secondary outcomes

Our primary outcome is protocol adherence, defined as >90% of participants adhering to their allocated treatment (that is, in the accelerated arm, starting RRT within 12 hours of eligibility; and in the standard initiation arm, starting RRT at least 12 hours following eligibility, considering patients who ultimately receive RRT). Compliance with protocol adherence will be captured for all randomized patients. For those allocated to the standard arm, we will ascertain the time of starting RRT (if started) relative to the time of fulfilling eligibility, and the indications for starting RRT. We will capture the specific reasons for non-compliance and crossover to starting RRT prior to 12 hours in those patients allocated to the standard arm.

Secondary outcomes include:

1. Proportion of eligible patients who are successfully enrolled in the trial (feasibility objective, >50% of eligible patients).

2. Proportion of enrolled patients for whom vital status and need for RRT can be determined at 90 days (feasibility objective, >95% of participants).

3. Serious adverse events potentially attributable to the study treatment, including death, vascular-access complications and complications associated with the delivery of RRT occurring up to day 14.

### Sample size

Our proposed sample size (n=100) will enable us to detect the primary feasibility target of 90% protocol adherence with a 95% CI of 79 to 96%. The lower limit of this CI would be the minimum acceptable in the proposed large definitive trial. Furthermore, assuming a normal distribution for the time from randomization to RRT initiation in our trial and SD estimated from a previous trial (SD 4.96) [11], we estimate that our sample size will have >95% power to detect a difference of at least 24 hours in time to RRT initiation between the accelerated and standard arms. Considering secondary endpoints, our sample size will permit calculation of CIs of 43 to 57% for the enrollment of 50% of eligible patients and 89 to 98% for achievement of 95% followup to 90 days.

### Analysis

Analysis will be intention-to-treat. The primary feasibility analysis will evaluate the proportion (95% CI) of all randomized patients adhering to their allocated treatment. The secondary feasibility analyses will evaluate the proportion (95% CI) of all screened eligible participants who are enrolled, and the proportion (95% CI) of enrolled patients having vital status and RRT dependence at 90 days ascertained. The secondary safety analysis will evaluate differences in the incidence of adverse events between the accelerated and standard groups. Tertiary analyses of patient-centered and health-resource utilization outcomes between the groups will be performed, including mortality through day 90, need for RRT through day 90, a composite of major adverse kidney events (MAKE), duration of ICU stay, duration of hospitalization and hospital readmission through day 90.

Descriptive statistics, boxplots and histograms will be used to analyze individual baseline variables by accelerated or standard arms. Normally or near normally distributed, non-correlated variables will be reported as means with SD and compared using the appropriate Student’s *t*-test. Non-normally distributed, non-correlated continuous data will be reported as medians with IQRs and compared using the Mann Whitney *U*-test. Non-correlated categorical data, including the tertiary analyses such as mortality and need for RRT, renal recovery and hospital mortality, will be reported as proportions and compared using Fisher’s exact test. Normally distributed correlated data will be analyzed by repeated measures analysis of variance (ANOVA). Non-normally distributed correlated data will be analyzed by the Friedman test. Correlated categorical data will be analyzed by generalized estimating equations. A *P*-value <0.05 will be considered significant. All statistical tests will be two-sided.

### Adverse events

Adverse events that are potentially attributable to the study treatment, including death, vascular-access complications (that is, hemorrhage, thrombosis, or infection) and complications associated with the delivery of RRT (that is, dialysis-associated hypotension, electrolyte abnormalities, and arrhythmias) will be documented during the first 14 days after randomization (Table 2). In addition, all serious adverse events that occur within 14 days after randomization are to be documented and reported, regardless of direct connection to the study. A serious adverse event is defined as: 1) any event that is fatal or immediately life-threatening, permanently disabling, severely incapacitating, or requires prolonged inpatient hospitalization, or 2) any event that may jeopardize the patient and requires medical or surgical intervention to prevent one of the outcomes listed above. We will track all adverse events until resolution or day 90, whichever comes first.

**Table 2:** Reportable adverse events

**Related to renal replacement therapy (RRT)**	**Related to central venous catheter (CVC)**
RRT-associated hypotension:	Hemorrhage at the site of CVC insertion:
Drop in blood pressure requiring one of: initiation of a vasopressor during RRT session, or need to escalate dose of a vasopressor during the RRT session, or premature discontinuation of RRT session, or any other intervention to stabilize blood pressure.	Bleeding requiring transfusion of ≥1 unit(s) of packed red blood cells and/or surgical intervention/repair within 12 hours following insertion
Severe hypophosphatemia:	CVC-associated bloodstream infection:
Serum phosphorus <0.5 mmol/L	Bacteremia in 2 blood culture sets with no proven alternative source for bacteremia or culture-positive recovery of the same organism from the dialysis catheter upon removal
Severe hypokalemia:	Ultrasonographically-confirmed thrombus attributed to CVC
Serum potassium <3.0 mmol/L	
Severe hypocalcemia:	Pneumothorax (for catheters placed in the internal jugular or subclavian positions)
Albumin-adjusted serum calcium <2.00 mmol/L or ionized calcium <1.00 mmol/L	
Allergic reaction	Hemothorax (for catheters placed in the internal jugular or subclavian positions)
Arrhythmia during dialysis:	Air embolism
New atrial (excluding sinus tachycardia or sinus arrhythmia) or ventricular arrhythmia that develops during RRT and was not present prior to initiation of RRT	
Seizure	Inadvertent arterial puncture at time of CVC insertion
Hemorrhage in patient receiving RRT with heparin-based anticoagulation	Other CVC-related adverse events

This table outlines the adverse events that are to be documented and reported during the first 14 days after randomization.

### Trial oversight

Steering Committee:the Committee will meet monthly by teleconference to review and address operational issues.

Coordinating Center:the AHRC of the Keenan Research Centre in the Li Ka-Shing Knowledge Institute of St. Michael’s Hospital, University of Toronto, will serve as the central trial coordination and data management center. AHRC will work closely with the study sponsor and Steering Committee to coordinate trial activities, and will be responsible for managing trial sites, obtaining regulatory approvals, safety reporting, maintaining and archiving trial documentation, sourcing and distributing trial materials, developing electronic case report forms, training trial staff, performing data validation and monitoring activities, performing statistical programming and analysis, and providing results of analyses.

Data and Safety Monitoring Board (DSMB): the three-member DSMB is comprised of experts in nephrology, critical care, and clinical research. The DSMB will meet by teleconference every 6 months while the trial is actively recruiting. The primary role of the DSMB will be to monitor adverse events among trial participants. The DSMB will assess whether these events are linked to participation in the trial or to any aspect of the study protocol. A DSMB charter has been drafted by the Steering Committee (see Additional file 6). The trial has been reviewed and endorsed by the Canadian Critical Care Trials Group (CCCTG).

## Discussion

The optimal timing of RRT initiation for severe AKI is currently unknown. Data from observational studies and recent systematic reviews [9,10,26-31] suggest that earlier commencement of RRT may benefit patients, but high-quality evidence to support this decision is currently lacking. Prior to starting a large, definitive RCT addressing this question, a pilot study is necessary to determine the feasibility of participant recruitment and protocol implementation in addition to assessing safety.

Strengths in the design of our pilot trial include carefully defined feasibility objectives, a clinically grounded pragmatic approach to enrollment and to the delivery of RRT, methods to reduce bias (allocation concealment, objective outcomes, and steps to ensure complete follow-up), recruitment from centers experienced in critical care and RRT research, and incorporation of alternate consent models. These steps will reliably inform decision-making regarding the design of the definitive trial. In addition, our inclusion criteria acknowledge that conventional metrics of kidney function poorly predict the need for RRT. Accordingly, a novel and unique aspect of our trial is the incorporation of a kidney-damage-specific biomarker, NGAL, in the patient selection process.

The design of this pilot RCT has limitations. First, the inclusion criterion of central venous pressure ≥8 mmHg does not reliably identify patients who are adequately volume resuscitated, but this measurement is more likely to be available than other functional hemodynamic assessments [32]. Second, although we have provided general recommendations that reflect prevailing clinical practice and are consistent with current clinical practice guidelines, we have not protocolized other aspects of RRT delivery, such as clearance and modality, in the absence of data confirming the clear superiority of a particular approach [33,34]. Although caregivers are not blinded in this trial, clinician awareness of treatment assignment in two recent RCTs of RRT in the ICU did not compromise study validity [12,25]. Third, it is possible that attending intensivists and nephrologists will have firm beliefs on the timing of RRT initiation and decline to allow their patients to be enrolled. However, a survey of Canadian intensivists and nephrologists (n=180) found that the large majority (93.7%) believed it would be ethical to randomize patients into such a trial [14].

RRT is an important and core life-support technology for critically ill patients with AKI, but it is also labor-intensive, expensive, and associated with complications. The timing of its initiation is a fundamental part of the RRT prescription, but there are no high-quality data to guide clinicians on when they make this decision [11]. The STARRT-AKI pilot RCT is a vital first step to filling this critical knowledge gap and better informing practice.

## Trial status

Recruitment is active.

## Abbreviations

AHRC: Applied health research centre; AKI: Acute kidney injury; CCCTG: Canadian Critical Care Trials Group; CRRT: Continuous renal replacement therapy; CVC: Central venous catheter; CVP: Central venous pressure; DSMB: Data safety monitoring board; IHD: Intermittent hemodialysis; NGAL: Neutrophil gelatinase-associated lipocalin; REB: Research ethics board; RCT: Randomized controlled trial; RIFLE: Risk injury, failure, loss, end-stage, renal failure; RRT: Renal replacement therapy; SDM: Substitute decision maker; SLED: Sustained low-efficiency dialysis; SOFA: Sequential organ failure assessment; STAART-AKI: Standard versus accelerated initiation of renal replacement therapy in acute kidney injury.

## Competing interests

Dr Bagshaw has consulted for Gambro Inc. Drs Bagshaw and Wald have received speaking fees and research funding from Alere Inc.

## Authors’ contributions

RW conceived the study, designed the trial, and helped draft the manuscript. SB conceived the study, designed the trial, and helped draft the manuscript. NA helped design the trial and draft the manuscript. OS helped design the trial and drafted the manuscript. KP helped design the trial, participated in study coordination, and helped draft the manuscript. MW helped design the trial and drafted the manuscript. All authors read and approved the final manuscript.

## Supplementary Material

Additional file 1 Site investigators. Click here for file

Additional file 2 Screening algorithm. Click here for file

Additional file 3 Site Health Research Ethics Boards. Click here for file

Additional file 4 Sample consent form. Click here for file

Additional file 5 Consent algorithm. Click here for file

Additional file 6 Data Safety Monitoring Board. Click here for file
