# Supplementary material for: Standard versus accelerated initiation of renal replacement therapy in acute kidney injury (STARRT-AKI): study protocol for a randomized controlled trial
Source: Trials. 2013 Oct 5;14:320. doi: 10.1186/1745-6215-14-320 (PMC3851593; doi:10.1186/1745-6215-14-320)
Supplement: Additional file 1 — Site investigators. [file 1745-6215-14-320-S1.docx]

**STARRT-AKI Site Investigators**

| **Centre** | **Investigators** |
| --- | --- |
| University of Alberta Hospital | Sean M Bagshaw, Constantine Karvellas, Noel Gibney |
| St. Michael’s Hospital | Ron Wald, Karen Burns, Jan Friedrich, David Klein, David Mazer, Orla Smith |
| Sunnybrook Health Sciences Centre | Neill KJ Adhikari, Michelle Hladunewich |
| London Health Sciences Centre (Victoria Hospital and University Hospital) | Matthew Weir, Andrew House, Amit Garg, Claudio Martin |
| St. Joseph’s Healthcare Hamilton | Mark Soth, Deborah Cook, Michael Walsh |
| University Health Network (both Toronto General Hospital and Toronto Western Hospital) | Margaret Herridge, Bob Richardson |
| Centre hospitalier universitaire de Sherbrooke | François Lamontagne, Jean-François Bilodeau |
| The Ottawa Hospital (both the General Campus and the Civic Campus) | Lauralyn McIntyre, Ted Clark, Swapnil Hiremath |
| Mount Sinai Hospital | Stephen Lapinsky |
