# Supplementary figures and images for: Standard versus accelerated initiation of renal replacement therapy in acute kidney injury (STARRT-AKI): study protocol for a randomized controlled trial
Source: Trials. 2013 Oct 5;14:320. doi: 10.1186/1745-6215-14-320 (PMC3851593; doi:10.1186/1745-6215-14-320)

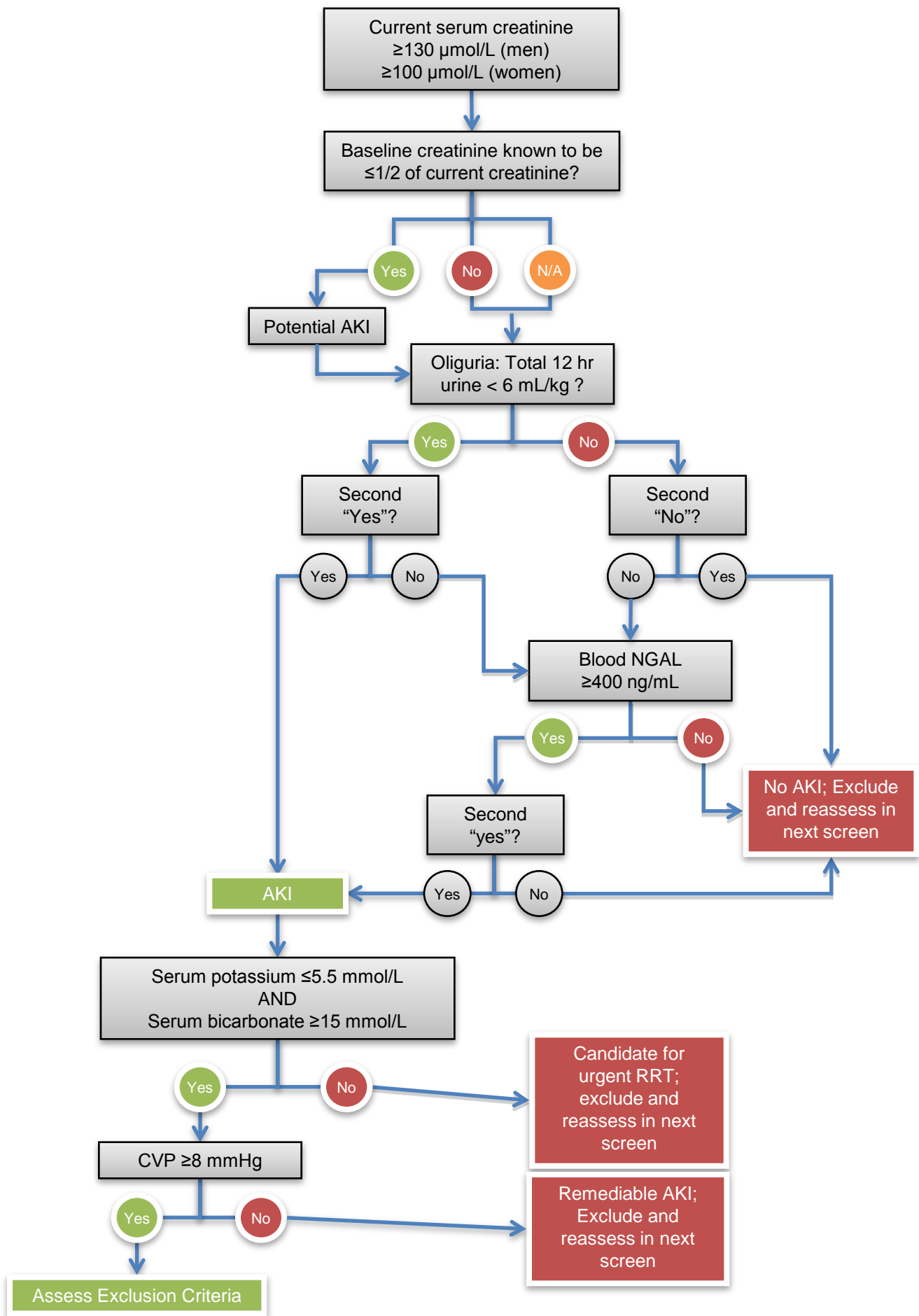

Supplement: Additional file 2 — Screening algorithm. [file 1745-6215-14-320-S2.pdf]
