# Supplementary material for: Standard versus accelerated initiation of renal replacement therapy in acute kidney injury (STARRT-AKI): study protocol for a randomized controlled trial
Source: Trials. 2013 Oct 5;14:320. doi: 10.1186/1745-6215-14-320 (PMC3851593; doi:10.1186/1745-6215-14-320)
Supplement: Additional file 3 — Site Health Research Ethics Boards. [file 1745-6215-14-320-S3.docx]

**STARRT-AKI Centre Health Research Ethics Boards:**

| **Centre** | **Ethical Body** |
| --- | --- |
| University of Alberta Hospital | University of Alberta Health Research Ethics Board |
| St. Michael’s Hospital | St. Michael’s Research Ethics Board |
| Sunnybrook Health Sciences Centre | Sunnybrook Health Sciences Centre Research Ethics Board |
| London Health Sciences Centre (Victoria Hospital and University Hospital) | University of Western Ontario Health Sciences Research Ethics Board |
| St. Joseph’s Healthcare Hamilton | Hamilton Integrated Research Ethics Board |
| University Health Network (both Toronto General Hospital and Toronto Western Hospital) | University Health Network Research Ethics Board |
| Centre hospitalier universitaire de Sherbrooke | Comité d’éthique de la recherche en santé chez l’humain |
| The Ottawa Hospital (both the General Campus and the Civic Campus) | Ottawa Hospital Research Ethics Boards |
| Mount Sinai Hospital | Mount Sinai Hospital Research Ethics Board |
