# Supplementary material for: Standard versus accelerated initiation of renal replacement therapy in acute kidney injury (STARRT-AKI): study protocol for a randomized controlled trial
Source: Trials. 2013 Oct 5;14:320. doi: 10.1186/1745-6215-14-320 (PMC3851593; doi:10.1186/1745-6215-14-320)
Supplement: Additional file 4 — Sample consent form. [file 1745-6215-14-320-S4.doc]

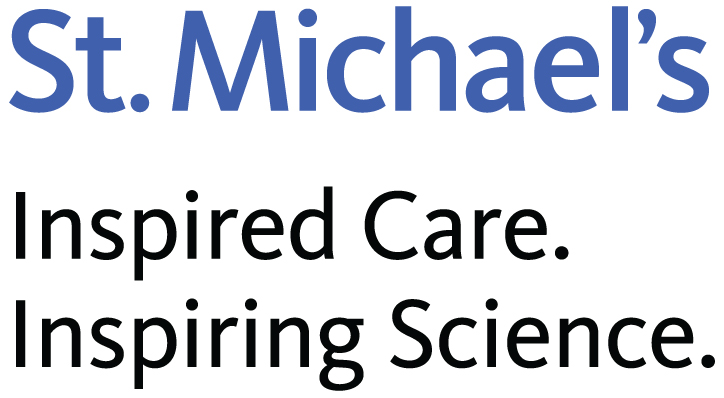
Letter of Information and Consent to Participate in a

Research Study

**Study Title: STandard versus Accelerated initiation of Renal Replacement Therapy in Acute Kidney Injury**

Short Study Title: The STARRT-AKI Study

**Principal Investigator:**

Ron Wald MDCM, MPH, FRCPC

Division of Nephrology, St. Michael’s Hospital

Tel: 416-867-3703 (Mon-Fri, 9 am-5 pm)

Tel: 416-864-5431 (all other times)

**Co-investigators:**

| Karen EA Burns, MD, MSc, FRCPC  Department of Critical Care Medicine,  Tel: 416-864-6060 x 3567 (Mon-Fri, 9 am-5 pm)  Tel: 416-864-5431 (all other times) | David Klein, MD MBA, FRCPC  Department of Critical Care Medicine  Tel: 416-864-6060 x 3566 (Mon-Fri, 9 am-5 pm)  Tel: 416-864-5431 (all other times) |
| --- | --- |
| Jan Friedrich, MD, MSc, DPhil, FRCPC  Department of Critical Care Medicine  Tel: 416-864-6060 x 3197 (Mon-Fri, 9 am-5 pm)  Tel: 416-864-5431 (all other times) | David Mazer, MD FRCPC  Department of Anesthesiology  Tel: 416-864-5825 (Mon-Fri, 9 am-5 pm)  Tel: 416-864-5431 (all other times) |
|  |  |
| Orla Smith, RN, BScN, MN  Department of Critical Care Medicine  Tel: 416-864-6060 x 3179 (Mon-Fri, 9 am-5 pm)  Tel: 416-864-5431 (all other times) |  |

**Study sponsor:** Ron Wald, MDCM MPH FRCPC

**Funding:** Canadian Institutes of Health Research

Alere (in-kind contribution of NGAL testing supplies)

Before deciding to participate in this research study, it is important that you read and understand the information contained in this consent form. This form provides all the information we think you will need in order to decide whether you (or your family member) wish to participate in the research study. If you have any questions after you read through this form, ask your doctor or one of the study personnel. You should not sign this form until you are sure you understand everything on this form. You may also wish to discuss your participation in this study with your family doctor, a specialist with whom you have had a longstanding relationship, a family member, or a close friend.

This consent form is intended for the patient who is eligible to take part in this study. However, if the patient is incapable of providing consent due to the severity of his/her illness, the consent of a relative or other authorized representative will be sought. If the participant becomes capable of providing consent during their ICU stay, informed consent will be sought from the participant. The pronouns “you” and “your” in this letter refer to the participant.

If the investigator will also be your treating doctor, this will be discussed with you.

**PURPOSE OF THE RESEARCH:**

You are being asked to consider taking part in this study because you have severe acute kidney injury (AKI) and may need renal replacement therapy, also known as dialysis, for this condition. AKI is the sudden loss of your kidneys' ability to get rid of excess fluid and waste material from your blood. When your kidneys lose their filtering ability, dangerous levels of fluid and waste products, including toxins, accumulate in your body. AKI is potentially life-threatening and common in the Intensive Care Unit (ICU). Often, patients with AKI require dialysis to replace the work normally carried out by the kidneys. This type of therapy uses a dialysis catheter (a large IV) to remove blood from the body and transmits it to a dialysis machine. The machine contains a filter which acts as an artificial kidney. Specifically, the filter removes waste products and excess fluid. The cleansed blood is then returned to the patient’s body through the catheter.

One of the major areas of uncertainty in AKI is, “When is the right time to start dialysis?” This question is the topic of this research study.

This preliminary (pilot) study is comparing two approaches to the timing of starting dialysis:

- accelerated (or early) initiation OR
- standard initiation.

This study will include 100 patients from at least 6 different hospitals across Canada. Your participation in this study will help the study doctors to assess whether the care teams can follow the protocols (set of steps) developed for the study and complete the necessary documentation and patient follow-up. If the pilot phase demonstrates that all of these are manageable, the investigators will proceed to a larger study that will test whether early or standard initiation of dialysis is better for improving patient survival and kidney recovery in critically ill patients with AKI. Information acquired from patients enrolling in the pilot phase may be included in the larger study.

**DESCRIPTION OF THE RESEARCH:**

If you agree to participate in this research study, you will have an equal (50/50) chance of being assigned to an accelerated (early) start of dialysis or standard initiation of dialysis. You will be assigned randomly (by chance, like the flip of a coin).

If you are randomized to the ***accelerated start of dialysis***, the goal will be to initiate dialysis as soon as possible. A temporary dialysis catheter (large IV) will be inserted into a large vein chosen by the critical care doctors. You will then be connected to a dialysis machine.

If you are randomized to ***standard initiation of dialysis*** and if your kidney function does not improve on its own, the decision to start dialysis will be guided by the usual laboratory values that are used by clinicians in everyday practice to start dialysis. Ultimately, the final decision will be made by your physicians, as would be the case if you were not participating in this study. The physicians will use their best judgment to decide when you need to start dialysis. The study team will also watch your bloodwork carefully and if they notice any features that suggest a need to start dialysis, they will notify your doctors. However, the final decision about whether and when to start dialysis will rest with the doctors who are caring for you.

As part of the study, we would also like to collect 0.5 mL (the equivalent of 1/10 of a teaspoon) of blood for testing of a substance called NGAL, if this has not been collected already. NGAL is likely to be elevated in patients with kidney damage and we would like to know whether NGAL helps predict how patients will progress over their course in hospital. After you have consented to the study but before any dialysis has started, the sample for NGAL will be taken from a tube that is already in your vein and you will not need any additional needles inserted in your skin to get this blood. The specific NGAL test being used in this study is an investigational test and has not been approved by Health Canada for use in standard practice.

Regardless of which group you have been assigned to, you will undergo the same procedures. The only difference between the two groups is the time you start receiving dialysis.

While on the study, the critical care doctors and nurses will care for you in the usual way. The research team will collect data from your medical records about your health status and the dialysis treatments that you receive for up to 14 days after you enter the study. Dialysis will be stopped if your critical care doctor and/or nephrologist judge(s) that your kidneys have recovered. After your dialysis has been discontinued and/or following discharge from the ICU, you will be followed by a member of the study team to determine your health status. This follow-up will end 90 days after your initial enrollment in the study. We will determine your health status at that time by reviewing your hospital chart, calling you at home and if necessary, by contacting you or your family doctor by telephone.

The study will involve collecting basic demographic (i.e. age, sex) information about you, as well as a review of your medical history including diseases/conditions that you have or have had in the past. Throughout the study we will collect medical information from your chart. We will be documenting lab results, medications, vital signs, and other data about the function of your organs. We will collect data on a daily basis for up to 14 days during your ICU stay. After you leave the ICU, we will document your date of discharge from the hospital.

POTENTIAL HARMS (INJURY, DISCOMFORTS OR INCONVENIENCE):

The risks of participation in this study are related to the dialysis procedure as well as to the catheter (IV tube) that needs to be inserted in order to perform dialysis. Some patients randomized to accelerated dialysis may have experienced improved kidney function spontaneously if dialysis had been delayed and, therefore, may not have needed dialysis. As such, these individuals would not have been exposed to the risks of dialysis and the insertion of the dialysis catheter in their vein. It is expected, however, that patients in both groups will eventually need dialysis (with the only difference between groups being the *timing* of dialysis initiation), thus the risks are similar in both groups. For most patients, participation in this study should not come with extra risk. Even in the unlikely situation that an adverse event occurs as a result of participating in this study, the events described below are mild-moderate in nature and can be fixed. In very rare circumstances, the consequences can be severe and even fatal. The added risks described below are most relevant to such patients.

Complications associated with dialysis include:

- A drop in blood pressure may occur in up 20% of treatments requiring the administration of intravenous fluid, medications or early halting of the dialysis treatment.
- Low phosphate values in the blood may occur in approximately 10% of patients; in rare cases, low phosphate values may cause muscle weakness. This is easily treatable with replacement of phosphate intravenously.
- Low potassium values may occur in less than 5% of cases; in rare cases, low potassium values may cause muscle weakness and heart rhythm disturbances. This is easily treatable with replacement of potassium intravenously.
- Allergic reaction to one of the components of the dialysis machine; this is extremely rare occurring in less than 1% of cases.
- Heart rhythm disturbance during dialysis may occur in 4% of cases.
- Seizures may occur in < 1% of patients receiving dialysis.

Adverse events potentially related to the central venous catheter used for dialysis:

- Bleeding at the site of catheter insertion in 1-2% of cases.
- Blood infection in 3% of cases.
- Symptomatic blood clot in 0.3% of cases.
- Air in the space surrounding the lung in 0.2 % of cases.
- Blood in the space surrounding the lung in < 0.1 % of cases.
- Air embolism in < 0.1 % of cases.
- Inadvertent insertion of the catheter into an artery instead of a vein in 0.5% of cases.

Due to the close monitoring given to patients in the intensive care until, occurrence of any of the above complications will be quickly recognized and in the vast majority of cases, the complication can be quickly reversed.

**POTENTIAL BENEFITS:**

It is possible, as has been suggested by several small studies, that earlier dialysis leads to better outcomes including improved survival. We will only know this for certain once the large study that we are planning is completed. However, patients randomized to early dialysis initiation in this pilot study may benefit if it turns out that early dialysis initiation is truly a better treatment strategy than the current standard of care.

**ALTERNATIVES TO PARTICIPATION:**

You do not need to participate in this study to receive treatment for your condition. There are other options available to you. In making your decision, you should bear in mind that being in the study is not a form of treatment and that being studied is not the same as being treated. If you choose not to participate, you will receive therapy for your AKI based on the usual clinical practice of the physicians’ caring for you.

**PROTECTING YOUR PERSONAL HEALTH INFORMATION:**

All persons involved in the study, including the study investigators, coordinators, nurses and delegates (hereby referred to as “study staff”), are committed to respecting your privacy. No other persons will have access to your personal health information without your consent, unless required by law. The study personnel and the study sponsor will make every effort to keep your personal health information private and confidential in accordance with all applicable privacy legislations, including the Personal Health Information Protection Act (PHIPA) of Ontario. The study personnel will access and look at your medical records and other personal health information and collect only the information they need for the study.

Personal health information is any information that could be used to identify you and includes your:

- name,
- address,
- date of birth.

Any personal identifying information (such as your name) will be “de-identified” by replacing your personal identifying information with a “unique study number”. The study coordinator and principal investigators at St. Michael's Hospital are in control of the study code key, which is needed to connect your personal health information to you. The link between the study number and your personal identity will be safeguarded by the St. Michael’s Hospital principal investigator.

To protect your privacy, data will be password-protected and securely stored. In addition, access to records and data will be limited to authorized persons and transmission of the data will be secure.

The study personnel will use your study information to conduct the study.

By signing this form, you are authorizing access to your medical records by the study personnel, the St. Michael’s Hospital Research Ethics Board and by applicable government regulatory authorities and/or authorized representatives of the study sponsor. Such access will be used only for the purpose of verifying the authenticity and accuracy of the information collected for the study, without violating your confidentiality, to the extent permitted by applicable laws and regulations. Alere (the company that is providing support for measurement of blood NGAL) will not have access to your medical records or personal health information.

All information collected during this study, including your personal health information, will be kept confidential and will not be shared with anyone outside the study unless required by law. You will not be named in any reports, publications, or presentations that may come from this study.

The study investigators will keep your study records securely stored for 10 years.

**STUDY REGISTRATION AND STUDY RESULTS:**

A description of this clinical trial will be available on *http://www.ClinicalTrials.gov.* This Web site will not include information that can identify you. Any results from this study will be published in a medical journal in a confidential manner.  Your name or any other identifying information will not be used in any summary or publication. If you are interested in obtaining the results/publication of this study, you should contact your study doctor.

**COSTS TO PARTICIPATION and REIMBURSEMENT:**

There are no costs to you should you decide to participate in this study. You will not be reimbursed nor be given money as a result of your participation.

**COMPENSATION FOR INJURY:**

If you suffer a physical injury as a direct result of the administration of the study procedures, medical care will be provided to you in the same manner as would ordinarily be provided. In no way does signing this form waive your legal rights nor relieve the investigators/sponsors/institutions involved from their legal and professional responsibilities.

**PARTICIPATION AND WITHDRAWAL:**

Participation in research is voluntary. You may refuse to participate, refuse to answer any questions, or withdraw from the study at any time with no effect on your or your family’s future medical care. If you choose not to participate, you and your family will continue to have access to customary care at St. Michael’s Hospital.

If you choose to withdraw from the study, the data you provided up to the point of termination (withdrawal) and information about your health status may still be used in the analysis. No further information, unless required for safety reasons, will be collected from you.

**RESEARCH ETHICS BOARD CONTACT:**

The study protocol and consent form have been reviewed by a committee called the Research Ethics Board at St. Michael’s Hospital. The Research Ethics Board is a group of scientists, medical staff, individuals from other professional backgrounds (including law and ethics) as well as members of the community. The committee is established by the hospital to review studies for their scientific and ethical merit. The Board pays special attention to the potential harms and benefits involved in participation to the research participant, as well as the potential benefit to society. If you have questions about the conduct of this study or your rights as a research subject you may contact Dr. Robert Hyland, Chair, Research Ethics Board, St. Michael’s Hospital at 416-864-6060 ext. 2557 during regular business hours.

**STUDY CONTACTS:**

You are invited to call Dr. Ron Wald the principal investigator at 416-867-3703, or any of the co-investigators at the telephone numbers listed at the beginning of this document. The investigators may also be reached through hospital locating at 416-864-5431 at all other times.

**STATEMENT OF CONSENT**

**Study Title: STandard versus Accelerated initiation of Renal Replacement Therapy in Acute Kidney Injury**

Short Study Title: The STARRT-AKI Study

The research study has been explained to me, and my questions have been answered to my satisfaction. I have been informed of the alternatives to participation in this study. I have the right not to participate and the right to withdraw without affecting the quality of medical care at St. Michael’s Hospital for me and for other members of my family. As well, the potential harms and benefits (if any) of participating in this research study have been explained to me. I have been told that I have not waived my legal rights nor released the investigators, sponsors, or involved institutions from their legal and professional responsibilities. I know that I may ask now, or in the future, any questions I have about the study. I have been told that records relating to me and my care will be kept confidential and that no information will be disclosed without my permission unless required by law. I have been given sufficient time to read the above information.

____________________ _______________________ _________________

Name of Participant Signature of Participant Date/Time (24h clock)

I have explained the study to the above Participant explained to the above Participant the nature and purpose, the potential benefits, and possible risks associated with participation in this research study. I have answered all questions that have been raised

____________________ _______________________ _________________

Name of Person Obtaining Signature of Person Obtaining Date/Time (24h clock)

Consent Consent

**SUBSTITUTE DECISION MAKER (SDM) STATEMENT OF CONSENT**

**Study Title: STandard versus Accelerated initiation of Renal Replacement Therapy in**

**Acute Kidney Injury**

Short Study Title: The STARRT-AKI Study

Participant’s Name (Print): ___________________

I acknowledge that the research study described above has been explained to me and that any questions that I have asked have been answered to my satisfaction. I have been informed of the alternatives to the above-named participant’s participation in this study, including the right not to consent to participation and the right to withdraw him/her without compromising the quality of medical care at St. Michael’s Hospital for him/her, myself or other members of our family(ies). As well, the potential risks, harms and discomforts have been explained to me and I also understand the benefits (if any) of his/her participating in the research study.

I understand that I have not waived his/her legal rights nor released the study doctors, sponsor, or involved institutions from their legal and professional duties. I know that he/she and I may ask now, or in the future, any questions we have about the study or the research procedures. I have been assured that records relating to him/her and his/her care will be kept confidential and that no information will be released or printed that would disclose personal identity without permission unless required by law. I have been given sufficient time to read and understand the above information.

____________________ _______________________

Name of Participant Relationship of Substitute Decision Maker to Participant

____________________ _______________________ _________________

Name of Substitute Signature of Substitute Date/Time (24h clock)

Decision Maker Decision Maker

I have explained to the above Participant the nature and purpose, the potential benefits, and possible risks associated with participation in this research study. I have answered all questions that have been raised

____________________ _______________________ _________________

Name of Person Obtaining Signature of Person Obtaining Date/Time (24h clock)

Consent Consent

I am the Principal Investigator responsible for the conduct of this study at St. Michael's Hospital and I have delegated the explanation of this study to this participant to ____________________ (name of person conducting the consent discussion).

____________________ _______________________ _________________

Name of Investigator Signature of Investigator Date

**STATEMENT OF CONSENT (PARTICIPANT CAPACITY REGAINED)**

**Study Title:** **STandard versus Accelerated initiation of Renal Replacement Therapy in Acute Kidney Injury**

Short Study Title: The STARRT-AKI Study

I understand that permission was given for me to participate in this study by ____________________while I was too sick to make my own decisions. The research study has now been explained to me, and my questions have been answered to my satisfaction. I have been informed of the alternatives to participation in this study. I have the right not to participate and the right to withdraw without affecting the quality of medical care at St. Michael’s Hospital for me and for other members of my family. As well, the potential harms and benefits (if any) of participating in this research study have been explained to me. I have been told that I have not waived my legal rights nor released the investigators, sponsors, or involved institutions from their legal and professional responsibilities. I know that I may ask now, or in the future, any questions I have about the study. I have been told that records relating to me and my care will be kept confidential and that no information will be disclosed without my permission unless required by law. I have been given sufficient time to read the above information. I will receive a copy of this consent form.

At this time, I am now able to make my own decisions and:

___ (initials) I ***consent*** to continue to participate in this study.

___ (initials) I ***do not consent*** to continued participation but ***agree*** to the retention data collected when I was unable to consent and to the collection of follow-up data until Day 90.

___ (initials) I ***do not consent*** to continued participation and ***do not agree*** to the retention of

the data collected when I was unable to consent and to the collection of follow-up data until Day 90.

________________ _______________________ _________________

Name of Participant Signature of Participant Date/Time (24h clock)

I have explained to the above Participant the nature and purpose, the potential benefits, and possible risks associated with participation in this research study. I have answered all questions that have been raised

____________________ _______________________ _________________

Name of Person Obtaining Signature of Person Obtaining Date/Time (24h clock)

Consent Consent

I am the Principal Investigator responsible for the conduct of this study at St. Michael's Hospital and I have delegated the explanation of this study to this participant to ____________________ (name of person conducting the consent discussion).

____________________ _______________________ _________________

Name of Investigator Signature of Investigator Date

**CONSENT FORM (DECLARATION OF ASSISTANCE)**

**Study Title:** **STandard versus Accelerated initiation of Renal Replacement Therapy in Acute Kidney Injury**

Short Study Title: The STARRT-AKI Study

The participant was assisted during the consent process as follows:

____ (initial) The consent form was read to the participant/substitute decision maker, and the person signing below attests that the study was accurately explained to, and apparently understood by, the participant/substitute decision maker and consent to participate was freely given by the participant/substitute decision maker.

____ (initial) The person signing below acted as a translator for the participant/substitute decision maker during the consent process. He/she attests that he/she has accurately translated the information for the participant/substitute decision maker, and believes that the participant/substitute decision maker has understood the information translated and consent to participate was freely given by the participant/substitute decision maker. The person signing below is not involved in the research study. S/he agrees to keep confidential all personal information of the study participant.

____ (initial) The person signing below was a witness to the consent process when a substitute decision maker was providing consent on behalf of the patient, he/she and attests that the information in the consent form was accurately explained to and apparently understood by the participant's substitute decision maker and consent to participate was freely given by the participant's substitute decision maker. The person signing below is not involved in the research study. S/he agrees to keep confidential all personal information of the study participant.

_________________________________ _____________________ ________________

Printed name of Person Assisting Signature of Person Date

____________________________________________

Relationship to Study Participant (specify)

Contact Information:

______________________________________________________________________________
