# Supplementary material for: Standard versus accelerated initiation of renal replacement therapy in acute kidney injury (STARRT-AKI): study protocol for a randomized controlled trial
Source: Trials. 2013 Oct 5;14:320. doi: 10.1186/1745-6215-14-320 (PMC3851593; doi:10.1186/1745-6215-14-320)
Supplement: Additional file 5 — Consent algorithm. [file 1745-6215-14-320-S5.pdf]

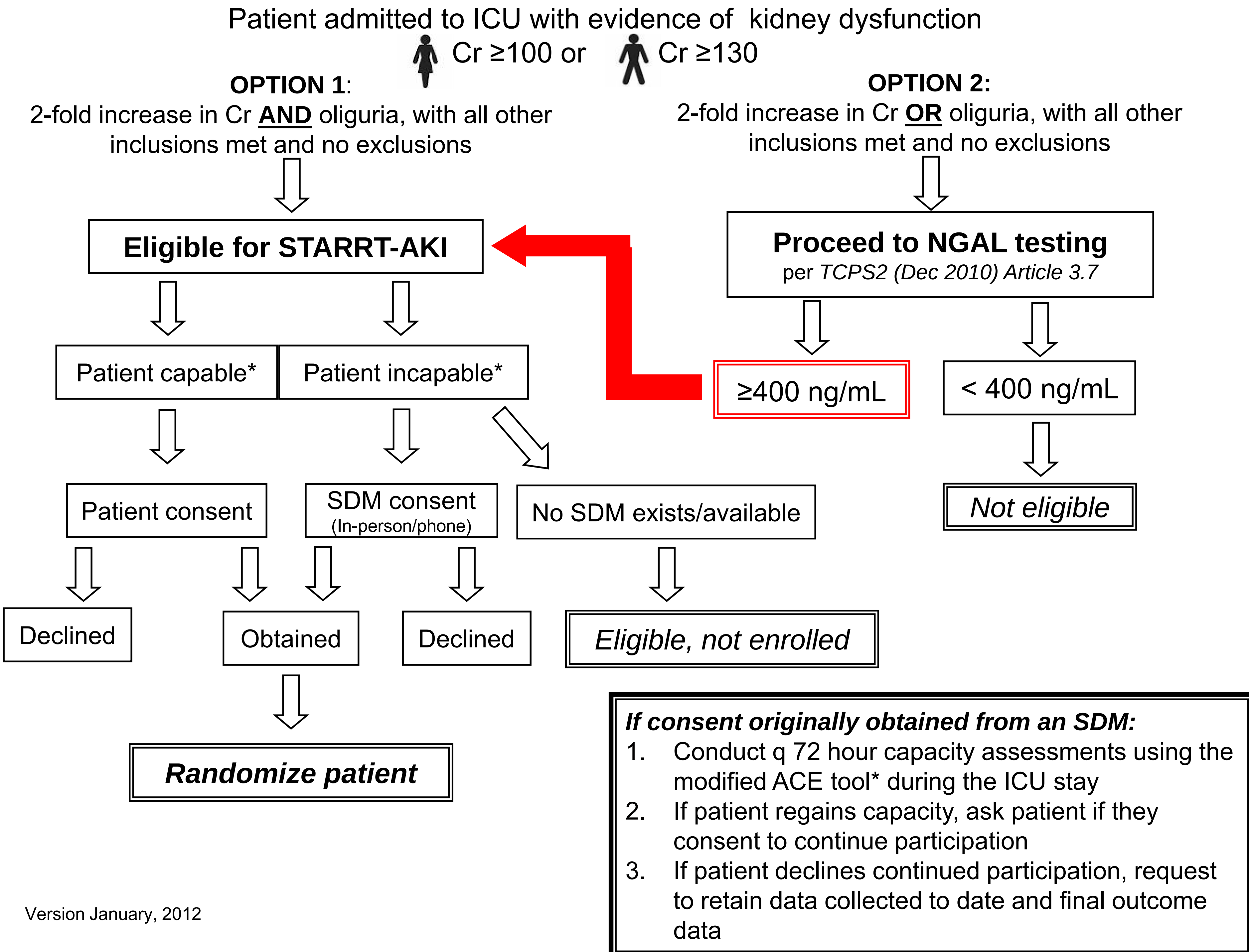
Patient admitted to ICU with evidence of kidney dysfunction
Cr ≥100 or
Cr ≥130
OPTION 1:
2-fold increase in Cr AND oliguria, with all other inclusions met and no exclusions
OPTION 2:
2-fold increase in Cr OR oliguria, with all other inclusions met and no exclusions
Eligible for STARRT-AKI
Proceed to NGAL testing
per TCPS2 (Dec 2010) Article 3.7
Patient capable*
Patient incapable*
≥400 ng/mL
< 400 ng/mL
Patient consent
SDM consent
(In-person/phone)
No SDM exists/available
Not eligible
Declined
Obtained
Declined
Eligible, not enrolled
Randomize patient
If consent originally obtained from an SDM:
1. Conduct q 72 hour capacity assessments using the modified ACE tool* during the ICU stay
2. If patient regains capacity, ask patient if they consent to continue participation
3. If patient declines continued participation, request to retain data collected to date and final outcome data
Version January, 2012
