# Supplementary material for: Standard versus accelerated initiation of renal replacement therapy in acute kidney injury (STARRT-AKI): study protocol for a randomized controlled trial
Source: Trials. 2013 Oct 5;14:320. doi: 10.1186/1745-6215-14-320 (PMC3851593; doi:10.1186/1745-6215-14-320)
Supplement: Additional file 6 — Data Safety Monitoring Board. [file 1745-6215-14-320-S6.doc]

**Data Safety Monitoring Board (DSMB) Charter**

**STandard versus Accelerated initiation of Renal Replacement Therapy in Acute Kidney Injury (STARRT-AKI)**

**February 22, 2012**

Principal Investigators (PIs):

1. Sean Bagshaw MD MSc, University of Alberta
2. Ron Wald MD MPH, St. Michael’s Hospital and University of Toronto

## Sean Bagshaw and Ron Wald are PIs conducting the STandard versus Accelerated initiation of Renal Replacement Therapy in Acute Kidney Injury (STARRT-AKI) randomized clinical trial. The PIs and selected co-investigators and the trial manager (Karen Pope, Matthew Weir, Neill Adhikari) constitute the Steering Committee (SC). The study is funded by the Canadian Institutes of Health Research and the University Hospital Foundation at the University of Alberta.

## AIMS and ROLES

## 1) Aims

## The Data and Safety Monitoring Board (DSMB) will act in an advisory capacity to the STARRT-AKI SC to monitor patient safety.

1. Specific roles

At 6-month intervals during the course of the trial, the DSMB will:

- Assess data quality, including timeliness and completeness
- Monitor compliance with the protocol
- Monitor participant recruitment, accrual and retention
- Review adverse event data
- Review protocol modifications made by the investigators
- Assess the impact and relevance of external data that may affect the safety of the participants or the ethics of the trial
- Monitor compliance with previous DSMB recommendations

The DSMB will monitor serious adverse events as they occur (see below), and at 6-month intervals.

## MEMBERSHIP

The DSMB consists of three members who are experts in nephrology and critical care. Members are independent of the investigators and have no financial, scientific, or other conflict of interest with the trial, as noted in written documentation on file with the Methods Centre at St. Michael’s Hospital.

Dr. Stuart Goldstein (University of Cincinnati, Cincinnati, USA) is the Chair, responsible for overseeing the meetings and the contact person for the DSMB. Other members include Drs Patrick Murray (University College Dublin, Dublin, Ireland) and Eric Hoste (Ghent University, Ghent, Belgium).

## RELATIONSHIP OF DMC TO PIs AND SC

The DSMB is independent of the PIs and SC with respect to recommendation made, but is supportive of the aims and methods of the trial. The DSMB serves in an advisory role. The DSMB, PIs, and SC will work collaboratively to ensure rigorous, safe, and timely conduct of the trial.

## MEETING OBJECTIVES and FREQUENCY

At the first meeting, members will

- discuss the protocol and suggest modifications at their discretion
- review, discuss, and approve mechanisms for transmitting serious adverse event information to the DSMB
- establish guidelines for calling emergency meetings of the DSMB

Meetings of the DSMB (in person or teleconference) will be held every 6 months at the call of the Chair, who may in addition call an emergency meeting at any time should questions of patient safety arise.

## MEETING FORMAT

Each meeting will start with an open session that may be attended by SC members and selected trial staff. Issues discussed will include conduct and progress of the study, including patient accrual, compliance with protocol, and any problems encountered. Patient-specific data and treatment group data may not be presented in the open session.

Only DSMB members will attend the closed session; others (e.g., study statistician) may attend by invitation. All safety data will be presented at this session. The discussion at the closed session is confidential.

After each meeting, the DSMB will recommend whether to (a) continue enrollment; (b) consult immediately with the PIs and SC with a view to terminating enrollment; or (c) request additional information before making a recommendation. Because this is a pilot study with no planned interim analyses, considerations that may influence the DSMB to recommend termination of enrollment include the number and nature of SAEs, methodological, or practical concerns. The DSMB will inform the PIs if, in their view, major safety issues have arisen that are likely to convince a broad range of clinicians, including those supporting the trial and the general clinical community, that on balance, continued use of one of the study interventions in a particular group or sub-group would be widely seen as unethical, BOTH for clinical care AND for any further investigation.

The DSMB will follow these guidelines in formulating a recommendation:

- the Chair will encourage consensus and all members will attempt to achieve consensus
- members will consider the ethical, scientific, statistical, practical, and financial implications for the trial in making recommendations.

Should the DSMB contemplate a recommendation to terminate the study, they will provide the SC with the opportunity to halt enrollment (without terminating the trial) and investigate any concerns during the halt period. If the DSMB subsequently decides to recommend termination, after considering the SC’s report of the issues raised, a vote of all DSMB members will be required. The DSMB will attempt to come to consensus before taking a vote. In the event of a divided vote, majority will rule and a minority report should be appended.

## SERIOUS ADVERSE EVENTS (SAEs)

## Definition

## A SAE is defined as:

## a) any event that is fatal or immediately life threatening, permanently disabling, severely incapacitating, or requires prolonged inpatient hospitalization, or

## b) any event that may jeopardize the patient and requires medical or surgical intervention to prevent one of the outcomes listed above

## All SAEs must be reported to the DSMB in aggregate form at least 10 days in advance of a scheduled meeting. However, those SAEs which the attending physician perceives may be directly related to enrolment in the STARRT-AKI Trial should be reported to the DSMB chair in an expedited fashion as per the procedures described below.

## *Note: Adverse events will be considered to be study-related if the event follows a reasonable temporal sequence from a study procedure and could readily have been produced by the study procedure. Adverse events are not study-related if they are related primarily to the underlying disease or to AKI and its sequelae.

## 2. Procedures for Reporting a SAE

## Each site research coordinator will liaise with the clinical team and review the medical records of study participants during the period of study RRT to identify potential SAEs. The site research coordinator will notify the site investigator and the local REB (according to local requirements) about each local SAE. Bedside clinicians will treat the study patient at their discretion.

## The site investigator has primary responsibility for the safety of individual study patients at his/her study site. Upon recognition of an SAE, the site research coordinator or site investigator will notify the STARRT-AKI Trial Methods Centre within 24 hours of becoming aware of the SAE, in accordance with standard SAE reporting requirements of regulatory authorities (e.g., Health Canada). The site research coordinator will complete the SAE form and any outstanding case report forms for that patient within 3 days. In addition, a copy of all relevant clinical notes will be forwarded to the Methods Centre, including all physicians’ and nurses’ notes, relevant diagnostic test results, and surgical and other intervention reports, within 3 business days of becoming aware of the SAE. These notes will be previewed at the Methods Centre to ensure that they do not contain sensitive or confidential patient information, in accordance with PHIPA requirements.

If the SAE is thought to be study-related and requires expedited reporting to the DSMB, the study manager (Karen Pope) will contact the PIs (Sean Bagshaw and Ron Wald) and the DSMB Chair (Dr. Stuart Goldstein) to alert them to forthcoming documentation regarding the SAE. Upon receiving all relevant clinical notes and case report forms from the site, research personnel at the Methods Centre will collate this material into a detailed report for distribution to the PIs and the DSMB Chair within 5 business days of the original notification to the Methods Centre.

After reviewing the clinical notes and CRFs, the DSMB chair will determine whether immediate input from other DSMB members is required and will contact them as needed. The DSMB will send their determinations to the PIs. Final determinations of the DSMB will be entered onto the relevant case report form, and into the database.

The DSMB will also review aggregate SAEs at 6-12 month intervals. At this time, the DSMB will recommend to the SC whether to (a) continue patient enrolment, (b) suspend enrolment until careful review by the SC, or (c) request additional information before making a recommendation.

## REPORTS

1. **Interim Reports:** Interim reports are distributed to the DSMB at least 10 days before a scheduled meeting. These interim reports will be numbered and provided in sealed envelopes within an express mailing package or by secure email as the DSMB prefers. The contents of the report are determined by the DSMB. Additions and other modifications to these reports may be directed by the DSMB on a one-time or continuing basis. Interim data reports generally consist of two parts:

Part 1 (Open Session Report) provides information on study aspects such as accrual, baseline characteristics, and other general information on study status, including protocol amendments.

Part 2 (Closed Session Report) will include safety data. The Closed Session Report is considered confidential and should be destroyed at the conclusion of the meeting.

**2. Reports from the DSMB:** A formal report containing recommendations for continuation or modifications of the study from the DSMB Chair will be sent to the full DSMB within 4 weeks of the meeting. It is the responsibility of the PI to distribute the formal DSMB recommendation report to all co-investigators and to ensure that copies are submitted to all site REBs.

The formal DSMB report should conclude with a recommendation to continue or to terminate the study. This recommendation should be made by formal majority vote after an attempt is made to reach consensus. A termination recommendation may be made by the DSMB at any time by majority vote. In the event of a split vote in favor of continuation, a minority report should be contained within the regular DSMB report. The report should not include unblinded data.

**Mailings to the DSMB:** Any concerns noted should be brought to the attention of the DSMB Chair or designated safety officer.

**Access to Interim Data:** Access to the accumulating endpoint data should be limited to as small a group as possible. Limiting the access to interim data to the DSMB members relieves the PIs of the burden of deciding whether it is ethical to continue to randomize patients and helps protect the study from bias in patient entry or evaluation.

## CONFIDENTIALITY

All materials, discussions and proceedings of the DSMB are completely confidential. Members and other participants in DSMB meetings are expected to maintain confidentiality.
